# Effect of PVA Fiber on the Mechanical Properties of Seawater Coral Sand Engineered Cementitious Composites

**DOI:** 10.3390/ma17061446

**Published:** 2024-03-21

**Authors:** Hongwei Han, Gongwen Gao, Yu Li, Dongxu Hou, Yudong Han

**Affiliations:** 1 School of Water Conservancy and Civil Engineering, Northeast Agricultural University, Harbin 150030, China; hanhongwei@neau.edu.cn (H.H.); 18045129438@163.com (G.G.); 18846771809@163.com (Y.L.); 18346238209@163.com (D.H.); 2 Heilongjiang Provincial Key Laboratory of Water Resources and Water Conservancy Engineering in Cold Region, Northeast Agricultural University, Harbin 150030, China; 3 Central Research Institute of Building and Construction, MCC Group Co., Ltd., Beijing 100088, China

**Keywords:** engineered cementitious composites (ECC), coral sand, uniaxial compression, splitting tensile

## Abstract

The physical and mechanical characteristics of seawater coral sand engineered cementitious composites (SCECC) were examined through uniaxial compression, three-point bending, and splitting tensile tests. The mechanical properties were scrutinized under varying fiber volume fraction conditions (V = 0%, 0.575%, 1.150%, 1.725%, and 2.300%). The experimental results indicated that the compressive strength, three-point bending strength, and split tensile strength of SCECC tended to increase with the rise in fiber volume fraction. The strengths attained their maximum values of 45.88, 12.56, and 3.03 MPa when the fiber volume fraction reached 2.300%. In the compression test, the compressive strength of the 7-day specimen can achieve more than 78.50% of that observed in the 28-day specimen. Three-point bending test has revealed that SCECC exhibits favorable strain-hardening and multi-crack cracking characteristics. Fracture patterns of SCECC exhibited variations corresponding to changes in fiber content, as illustrated by their load–deformation curves, the addition of PVA fibers can change the damage mode of cementitious composites from brittle to ductile. The fracture energy of SCECC further attests to its elevated toughness. This is due to the fact that the fibers delay the formation of microcracks and prevent crack expansion, thus significantly increasing the deformability of the material. By verifying its strength, deformability, fracture energy, and other key performance indicators, the feasibility of SCECC in coastal construction projects has been clarified. The successful development of SCECC provides an innovative and high-performance option for the construction of future island projects.

## 1. Introduction

Coral concrete represents an innovative building material created from coral sand, cement, admixtures, and mineral admixtures, all blended according to a specific mixing ratio [1]. It has already showcased benefits such as reduced construction timelines and lower costs in defense and maritime infrastructure development [2,3]. Nevertheless, coral concrete exhibits a degree of brittleness and susceptibility to cracking [1,4]. And these cracks, often on a millimeter scale, can readily evolve into channels facilitating the erosion of harmful ions. Engineered cementitious composites (ECC) are developed to solve the problems of concrete brittleness and susceptibility [5,6]. However, the high price of grinding and transportation of the fine silica sand used in ECC limits its engineering applications. In this study, we attempt to use seawater and coral sand to prepare SCECC instead of freshwater and silica sand in traditional ECC to reduce the cost of ECC production.

Some scholars have applied principles from fracture mechanics and micromechanics, along with a performance-driven design approach, to integrate short-cut fibers into cement, mineral admixtures, fine aggregates, and high-performance admixtures [7]. It is aimed to achieve fiber-reinforced cementitious composites characterized by two key features by designing and optimizing the fiber, matrix, and fiber–matrix interfaces: multi-slit cracking and strain hardening [8,9]. The addition of fibers ensures the stable expansion of ECC cracks and obtains multi-crack cracking characteristics. In the failure process of ECC, numerous fine cracks typically appear, with a crack width generally falling within the range of 100 µm [10,11,12]. This characteristic proves effective in preventing the infiltration of harmful substances. Even if multiple cracks are formed, the specimen can withstand higher loads, which exhibits significant toughness [13,14]. Initially, PE fibers were used to produce ECC [15], but their high cost made it difficult to promote the ECC material. Later, some scholars used polyvinyl alcohol (PVA) instead of PE fiber [16], which greatly reduced the cost of ECC materials. PVA fiber has high tensile strength and modulus of elasticity, which can effectively improve the toughness and impact resistance of cementitious materials. The tensile properties of PVA fiber-reinforced ultra-high toughness cementitious composite (UHTCC) were investigated [17]. The composite shows ultra-high ultimate tensile strain capacity above 4% under uniaxial loading, which is hundreds of times larger than the tensile strain observed in ordinary concrete [18,19]. Zhang [20] developed more cost-effective ECC materials suitable for civil infrastructure applications with low-cost type-C PVA fibers. The results of this study showed that its compressive strength could be up to 45 MPa and tensile strength could be up to 4.41–5.56 MPa.

Utilizing industrial wastes to substitute a portion of the cement in the ECC preparation process, such as fly ash, slag, lithium slag, and red mud slag, markedly diminishes energy consumption in the raw material production phase [21,22,23]. This aligns with the intention to promote environmentally sustainable development [24]. Nevertheless, the primary source of sand in ECC is typically river sand, a non-renewable resource. The over-exploitation of river sand can result in substantial price hikes, a reduction in sand reserves, and have a significant environmental impact [25]. Moreover, fine-grained river sand and freshwater pose potential challenges in certain construction projects, particularly in instances such as island projects situated far from the mainland [26,27]. The logistical constraints of transporting materials from abroad may lead to uncertainties in project schedules.

Due to the finite nature of this resource and its environmental impact, the urgent need for alternative materials is particularly prominent. Research has been conducted to explore the use of local materials in preparing ECC [28,29]. Using seawater and coral sand as the primary components of SCECC materials can fully leverage the advantages of ECC, including high ductility, durability, blast, and impact resistance while fully using marine resources. This minimizes reliance on traditional sand and freshwater. It also avoids the time and economic costs associated with transporting raw materials from the interior. Coral sand is extensively found in offshore islands and reefs, boasting abundant reserves and an essentially limitless supply of seawater [30,31]. Derived from the remains of coral reef organisms, coral sand is a sediment primarily composed of more than 96 percent calcium carbonate. Although the coral sand and seawater may help improve the pore structure and interfacial transition zone [32], the major factor restricting the use of coral concrete remains the chloride corrosion due to the inherent porous structure of corals and plenty of chloride ions from itself and seawater [33]. It has been shown that the incorporation of fly ash can reduce the drying shrinkage of coral concrete and improve the carbonation resistance, anti-permeability, and chloride penetration resistance of coral concrete [34].

SCECC not only signifies technological breakthroughs but also contributes to promoting environmentally sustainable development in island construction. This initiative is anticipated to address the deficiencies in the performance of current seawater coral aggregate concrete and establish a robust foundation for the construction of high-performance island engineering structures.

Building upon the aforementioned study content, this paper formulates a novel engineered cementitious composite utilizing coral sand and seawater. The objective is to examine the influence of fiber content on the mechanical properties of SCECC. Examined the influence of fiber content on the compressive strength, deformation capacity, flexural strength, fracture energy, multi-crack characteristics, and strain-hardening properties of SCECC through the execution of the uniaxial compressive test, three-point bending test, and split tensile test on specimens.

## 2. Materials and Methods

### 2.1. Materials and Preparation of SCECC

**Figure 1:**
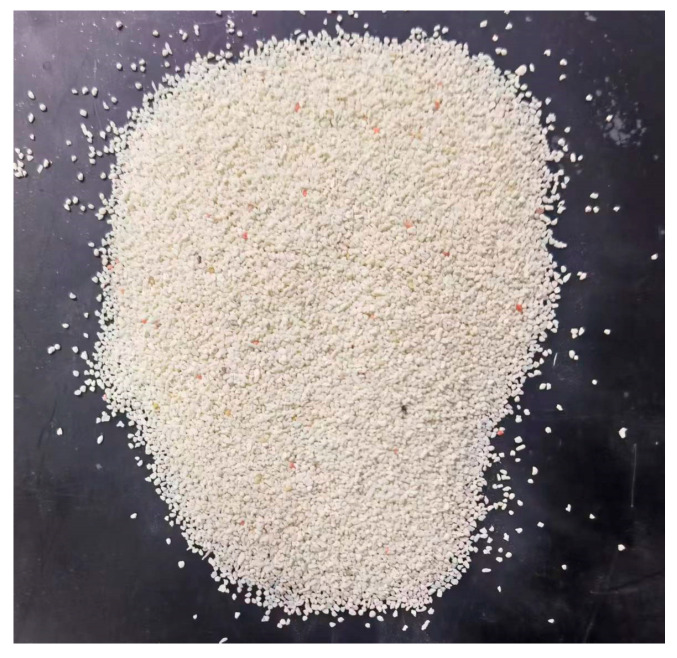
Coral sand from a tropical sea.

Based on the literature [35], the design mix ratios for this study are shown in Table 1. The specific mix proportions utilized in this study are outlined in Table 1. The basic materials for SCECC in this study include grade 42.5 ordinary Portland cement, Class Ⅰ fly ash, coral sand, seawater, PVA fiber, and superplasticizer. Among them, P·O 42.5 cement and fly ash are produced by Yatai Group Harbin Cement Co., (Harbin, China) and Hengyuan New Material Co. (Henan, China) respectively. As shown in Figure 1, the fine aggregate is coral sand collected from a tropical sea. Its chemical composition is shown in Table 2. Using such a small aggregate size reduced the size of the weak interface between the aggregate and the cement. A smaller aggregate also reduces the fracture toughness of the matrix for crack initiation and work of fracture during the steady-state crack propagation, both of which are desirable for composite ductility according to micromechanics [36]. Table 3 presents the aggregate grading parameters of ECC mixtures. The composition of simulated seawater, according to the seawater inspection report of Sanya Construction Engineering Quality Inspection Center, is shown in Table 4. The physical and mechanical properties of PVA fibers manufactured by Kuraray Corporation (Kurashiki, Japan) are shown in Table 5. Polycarboxylate superplasticizer for offshore concrete is provided by Tianjin Yejian Special Materials Co., Ltd. (Tianjin, China).

**Figure 2:**
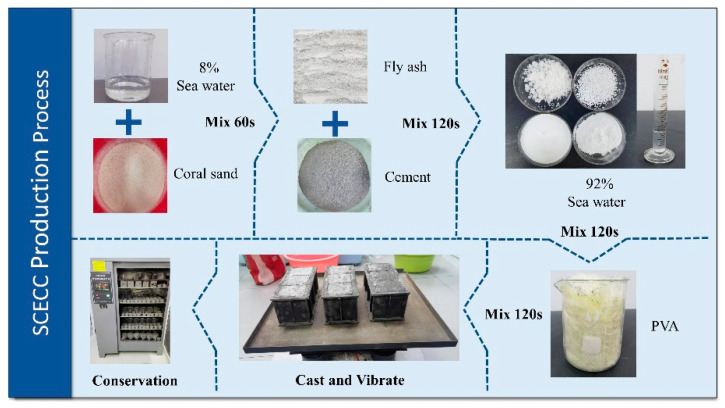
Fabrication process of SCECC specimens.

Figure 2 illustrates the fabrication process of SCECC specimens, depicting detailed steps in the procedure. Firstly, place the measured aggregate in a mixing tank and add 8% of the total water required for mixing, then stir for 1 min to achieve uniform pre-wetting of the aggregate. Next, combine cement and fly ash and introduce the mixture into the mixing pot. Stir the components for 2 min to ensure a thorough and even blending of the cementitious material with the aggregate. Pour the remaining mixed water and water-reducing agent into the mixing tank, and continue the mixing process for another 2 min to obtain a uniform slurry. Introduce all the PVA fibers into the mixing tank and mix for an additional 2 min to ensure the uniform distribution of the fibers within the cement matrix. After achieving a homogeneous mixture through stirring, pour it into the oiled molds. Subsequently, place the molds onto the vibrating table for 1 min, followed by smoothing the mixtures with a spatula. Cover the samples with plastic film and allow them to sit at room temperature for 24 h before demolding to prevent moisture loss. Finally, before the test, place the demolded specimens in a standard curing box with a temperature of 20.0 °C and a relative humidity of 95% for 7 d, 14 d, and 28 d. The curing box used in this study is produced by Hebei Xinmeihua Text Instrument Co., Ltd. (Cangzhou, China).

### 2.2. Test-Scheme Design

#### 2.2.1. Uniaxial Compression Test

**Figure 3:**
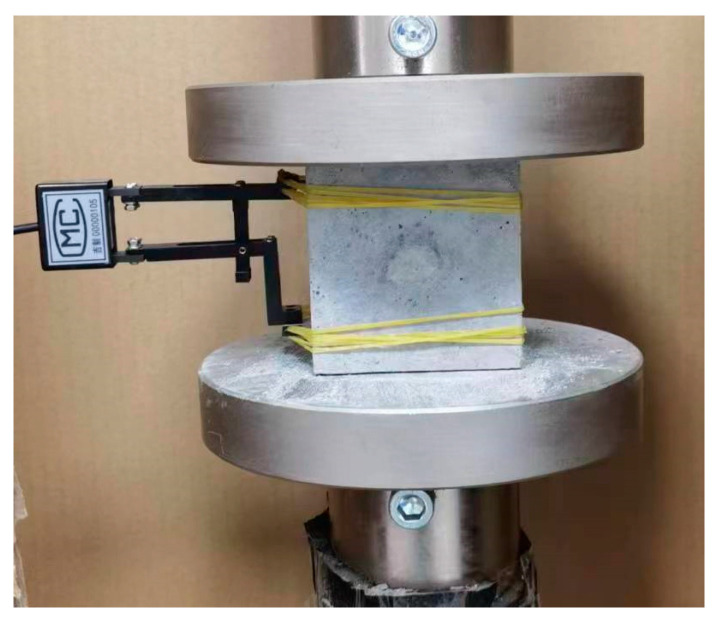
Compressive strength test specimen.

Five cubic blocks were cast for each set of specimens, each with a side length of 70.7 mm. An electronic compression testing machine with a measurement range of 300 kN, which was manufactured by Shandong Wanchen Instrument and Equipment Co., Ltd. (Jinan, China), was used as the loading equipment. To obtain the entire process of compression behavior, displacement-controlled loading was adopted, with a loading rate of 0.5 mm/min. A clip-on gauge with a gauge length of 50 mm produced by Changchun Kexin Experimental Instrument Co., Ltd. (Changchun, China) was installed on the side to measure the deformation of the specimen during compression. It was able to reduce the effect of the end restraint provided by the rigid loading plate during compression, as shown in Figure 3.

#### 2.2.2. Three-Point Bending Test

**Figure 4:**
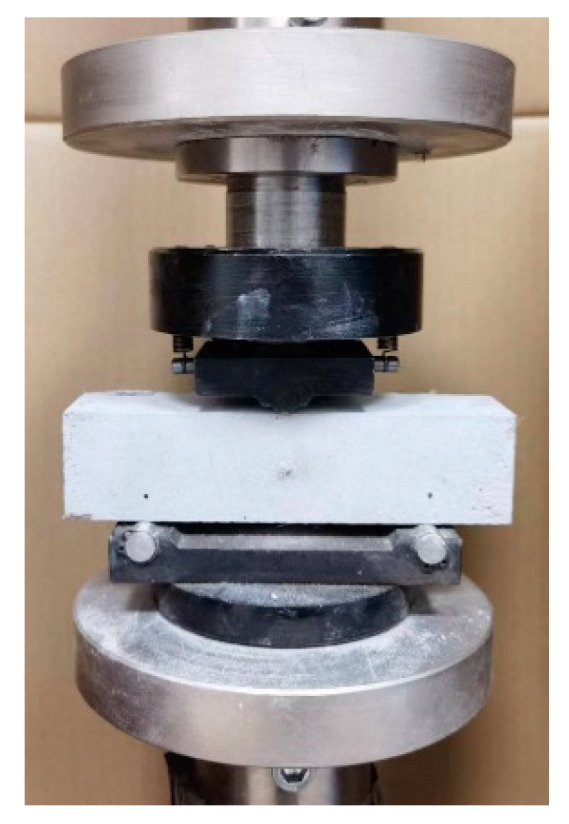
Three-point bending test loading device.

To elucidate the influence of fiber content on matrix toughness, the three-point bending test was conducted. Six prismatic beams measuring 160 mm (length) × 40 mm (width) × 40 mm (height) were prepared for the three-point bending test, featuring a support span of 100 mm, as illustrated in Figure 4. This test adopts the displacement-controlled loading mode of 0.5 mm/min. Record the load-acting displacement, load, and time of the universal testing machine.

According to the calculation Equation (1) recommended in the literature [37], the three-point bending strength *R_f_* can be calculated.

(1) $$R_{f}=\frac{3Fl}{2bh^{2}}$$

where *R_f_* is the bending strength (MPa), *F* is the maximum load (kN), *l* is supports distance (mm), *b* is the width, and *h* is the height of the specimen (mm).

The calculation for bending strain is as follows:

(2) $$\varepsilon_{f}=\frac{6hf}{L^{2}}$$

where *ε_f_* is the bending strain, *f* is the load-acting displacement (mm).

Fracture energy refers to the energy consumed per unit area of the SCECC material during crack expansion. The calculation for fracture energy is as follows [38]:

(3) $$G_{F}=\frac{W+mg\delta }{A}$$

where *G_F_* is the fracture energy (J/m^2^), *W* is the work carried out by the SCECC in the three-point bending test (J), m is the mass of the specimen between the supports (kg), *g* is the acceleration of gravity (9.8 m/s^2^), *δ* is the final displacement at failure (m), *A* is the actual area of the fracture surface of the specimen (m^2^).

#### 2.2.3. Splitting Tensile Test

**Figure 5:**
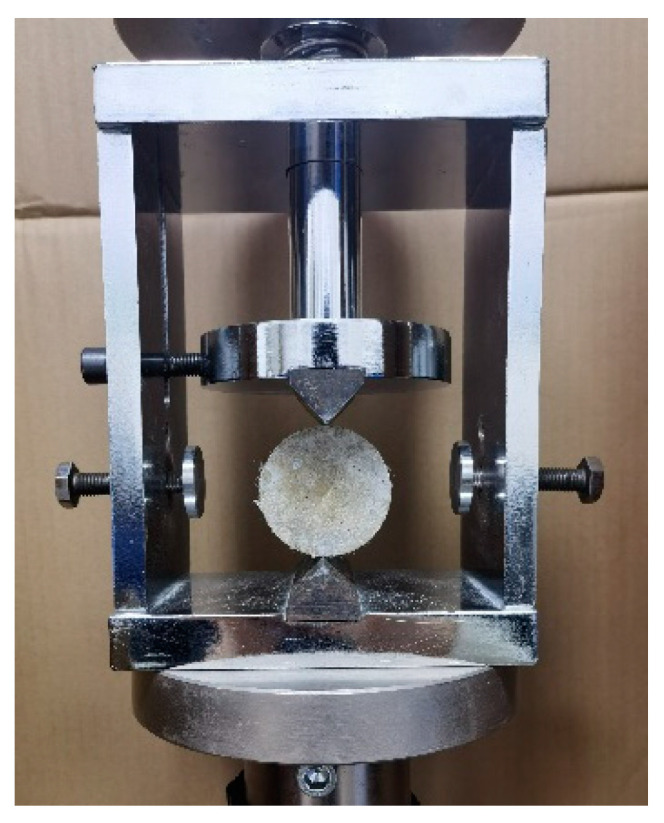
Splitting tensile test angular fixture.

The splitting tensile test is employed to assess the tensile properties of the SCECC specimens. To ensure that the splitting tensile test approximates the linear elastic state before crack initiation, an angle clamp is employed in this study, as depicted in Figure 5. After curing, 5 cylinder specimens were tested, which were 50 mm in diameter and 25 mm in thickness. The test adopts a displacement-controlled loading mode of 0.12 mm/min. A computerized and accurate data acquisition system was utilized to record displacement and load.

The splitting tensile strength was determined using Equation (4) [39]:

(4) $$\sigma_{t}=\frac{2F}{\pi Dt}$$

where *σ_t_* is the splitting tensile strength (MPa), *F* is the maximum load applied to the specimen (kN), *D* is the diameter (mm), and *t* is the thickness (mm).

## 3. Results and Discussion

### 3.1. Uniaxial Compressive Results Analysis

**Figure 6:**
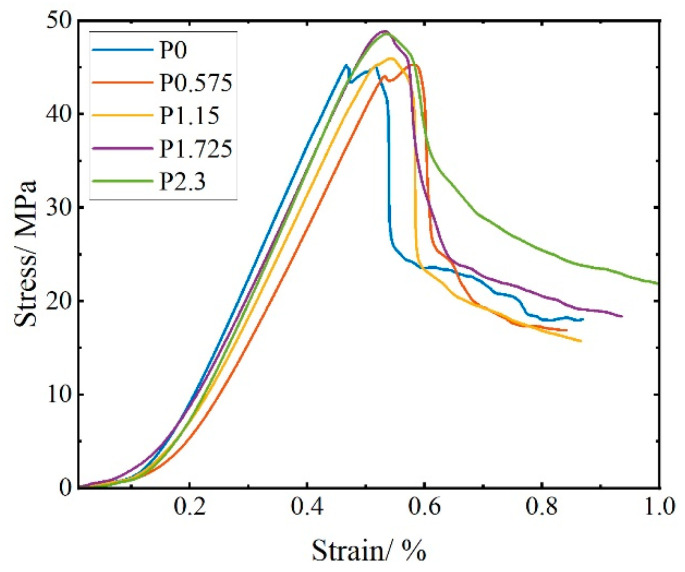
Uniaxial compressive stress–strain curves of SCECC.

Figure 6 shows the stress–strain curve under uniaxial compressive. Four stages can be distinguished from the curves [3]: initially, a compacting stage, followed by elasticity, then the cracking stage, and, finally, the failure stage. In the compacting stage, because there are fine cracks inside the concrete that close under the action of external forces, the curve shows a slow upward trend. In the elastic stage, the stress and strain exhibit a linear relationship, and the rate of stress increase is significantly accelerated. In the cracking stage, cracks emerge for the first time in the specimen, with the PVA fiber serving as a bridge to impede crack expansion. Plastic deformation becomes predominant. The strain at the peak point of the stress–strain curve is called compressive strength, and the corresponding strain is referred to as the peak strain. In the failure stage, the stress–strain curve drops suddenly. The bridging capacity was unable to be performed from the PVA fiber, resulting in multiple cracks and large deformation. It is worth noting that the lower the fiber content, the faster the stress decreases. The stress of the P-0 sample decreases almost like a cliff.

**Figure 7:**
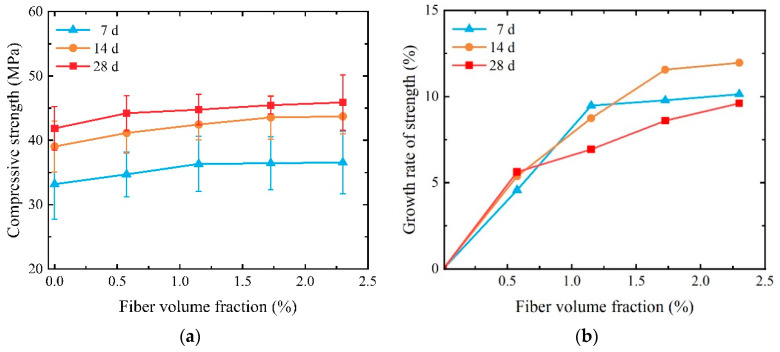
Uniaxial compressive strength of SCECC with different fiber volume fractions and ages: (**a**) compressive strength; (**b**) the growth rate of strength.

The compressive strength is affected by age, that is, the compressive strength increases as the concentration age increases, as shown in Figure 7. The compressive strength of the 7-day specimen can achieve more than 78.50% of that observed in the 28-day specimen. The fastest growth in compressive strength is found to be 83.21% for the specimen with *V_f_* = 1.150% (P-1.150), which demonstrated significant early strength. There are two reasons for this high early strength. First, SCECC is produced using seawater, which contains a large number of inorganic salts, and the chloride-ion content is significantly higher than that of river water. The chloride-ion content produces an acceleration of the cement setting and early hardening of the SCECC [40,41]. On the other hand, coral sand has a water absorption rate of 7%, and the porous microstructure of coral sand performs the function of ‘‘water bag”. It absorbs and reserves a certain amount of water from the SCECC slurry and releases the water in the hydration and hardening process, which also enhances the early strength of SCECC [42,43].

As shown in Figure 7b, the compressive strength of SCECC shows an increasing trend alongside the increase in PVA volume fraction, and the rate of increase decreases gradually. The compressive strength is found to be 44.21 MPa for the 28-day specimen with *V_f_* = 1.150% (P-1.150), and this is 5.62% more than the specimen with *V_f_* = 0%, respectively. When the fiber volume fraction is equal to or greater than 0.575, the strength growth rate of the 28-day specimen experiences a notable slowdown. The chaotic distribution of fibers makes it form a fiber network inside the matrix, and the tensile fibers can inhibit the development of fine cracks to a certain extent during the compressive damage process of SCECC specimens, thus making the compressive strength of SCECC increase. The compressive strength does not show a continuous and rapid increase with the augmentation of the fiber volume fraction. This phenomenon occurs because there is an optimum value for the PVA fiber volume fraction. When an excess of fiber is added, it is not easily dispersed uniformly. The aggregated fibers tend to form a weak interface with the cement paste, thereby failing to enhance the mechanical properties of SCECC. The compressive strength can reach a maximum of 45.88 MPa, which is greater than the coral concrete compressive strength [1], but relative to the UHDCC (max. 121.5 MPa) still has a certain gap [44]. This suggests that moderate-strength SCECC can be prepared using coral sand. However, higher strength grades of SCECC need to be further investigated.

**Figure 8:**
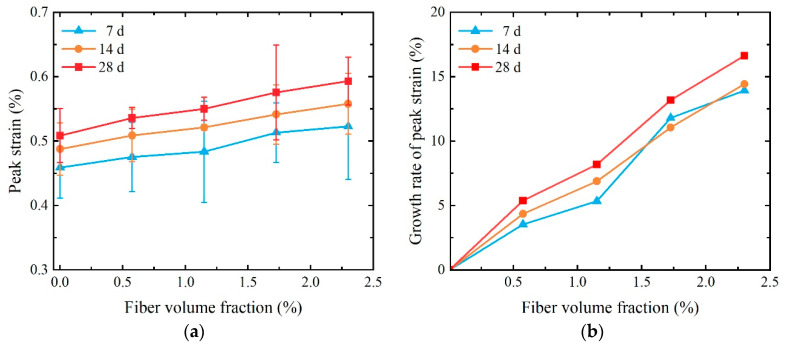
Uniaxial compressive strain of SCECC with different fiber volume fractions and ages: (**a**) peak strain; (**b**) the growth rate of peak strain.

Figure 8 shows the peak strain of SCECC with five different fiber volume fractions, with values ranging from 0.46% to 0.59%. The peak strain is affected by age; that is, the peak strain increases as age increases. The peak strain of the 7-day specimen can reach over 87.88% of that observed in the 28-day specimen. As illustrated in Figure 8b, the peak strain of SCECC exhibits an increasing trend with the rise in PVA fiber volume fractions. The peak strain is found to be 0.59% for the 28-day specimen with *V_f_* = 2.300% (P-2.300), and this is 16.64% more than the specimen with *V_f_* = 0% (P-0). This trend arises from the random distribution of PVA fibers in the sample, creating an overlapping network structure. This network structure effectively inhibits crack development, leading to an increase in the peak strain of SCECC.

### 3.2. Three-Point Bending Test Results Analysis

**Figure 9:**
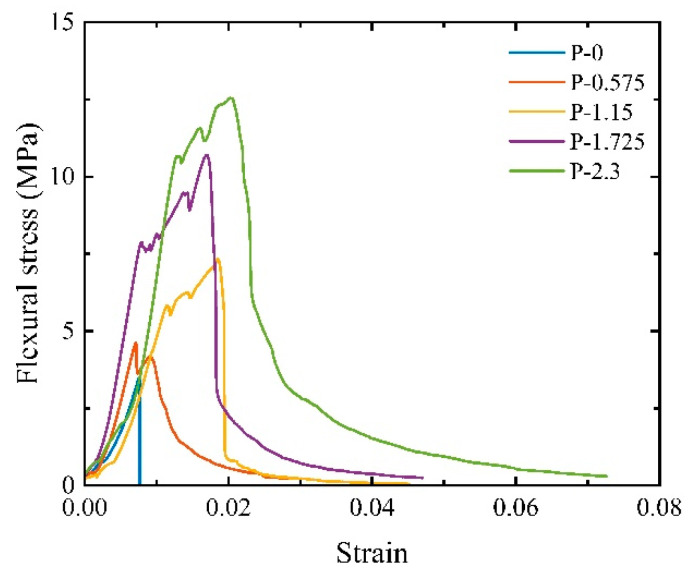
Three-point bending stress–strain curves of SCECC.

The typical stress–strain curve of the three-point bending test is illustrated in Figure 9. It shares similarities with the uniaxial compression stress–strain curve in that they both consist of four stages.

In the strain-hardening stage, following the initiation of the first fine crack in the lower tensile zone of the specimen, the bridging capacity of the PVA fibers effectively controls the extension of the crack, ensuring that the material does not undergo immediate failure. Simultaneously, stress is redistributed, resulting in the formation of a second fine crack adjacent to the first one, and so forth, eventually leading to the stable expansion of the crack. As a result, the material’s deformation capacity is significantly increased, and approximately 50% of the material deformation occurs at this stage. The appearance of each fine crack is accompanied by stress release within the material, causing a steep stress drop in the stress–strain curve. However, the specimen’s load-carrying capacity is regained as the stress is redistributed.

**Figure 10:**
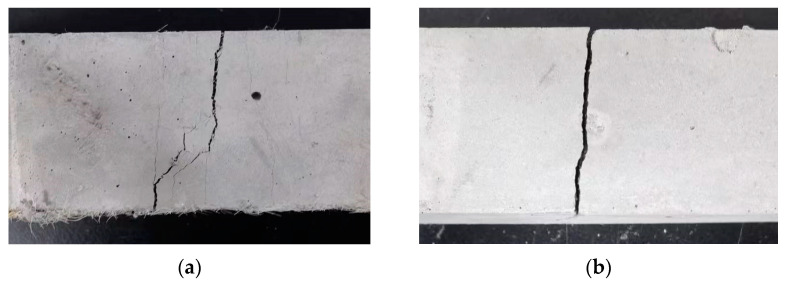
Failure mode of the three-point bending test specimen: (**a**) *V_f_* = 1.725%; (**b**) *V_f_* = 0%.

As shown in Figure 10, the specimen after failure maintains a single unit due to fiber action. However, the specimen with *V_f_* = 0% lacked the strain-hardening stage. After the specimen reached the point of initial cracking, cracks suddenly appeared and rapidly propagated through the entire specimen with further loading, leading to the ultimate failure of the specimen.

It is worth mentioning that for the specimens with *V_f_* = 0.575%, even though fibers were added, the load-carrying capacity of the specimen decreased after reaching the initial cracking point. While the fiber plays a bridging capacity, the amount of fiber doping is insufficient to induce multiple cracks in the specimen after stress redistribution. Therefore, the strength of the cracked specimen cannot reach the initial cracking strength.

**Figure 11:**
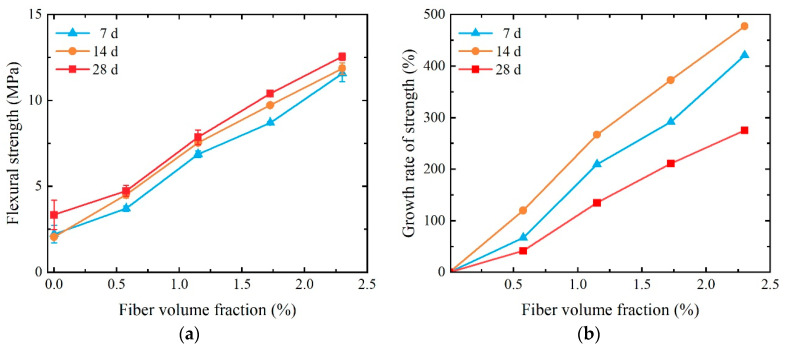
Three-point bending strength of SCECC with different fiber content and ages: (**a**) three-point bending strength; (**b**) the growth rate of strength.

As depicted in Figure 11, the bending strength of SCECC tends to rise with an increase in PVA fiber volume fraction and age. The bending strength of the 28-day specimen with *V_f_* = 2.300% (P-2.300) reaches 13.43 MPa, indicating a 275.30% increase compared to the 28-day specimens with *V_f_* = 0% (P-0). This suggests that the PVA fibers effectively bridge cracks in the specimens, maintaining the overall strength of the specimen. The flexural strength of SCECC is up to 13.43 MPa, which is not significantly different from that of the ECC studied by Özkan [45].

**Figure 12:**
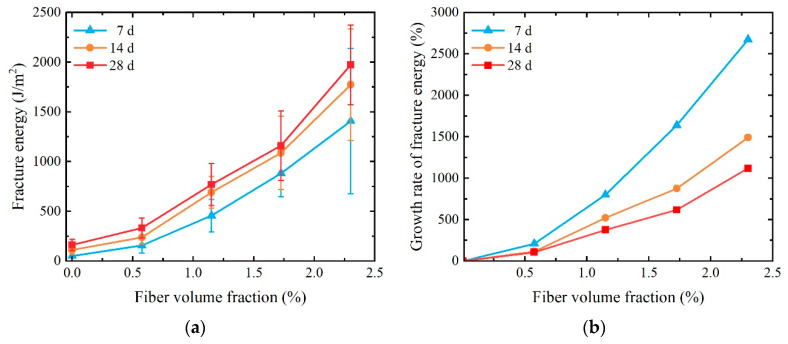
Fracture energy of SCECC with different fiber content and ages: (**a**) fracture energy; (**b**) the growth rate of fracture energy.

The fracture energy of the five different fiber volume fractions can be obtained by Equation (3), as listed in Figure 12. It is found that the fracture energy of SCECC specimens increases as the age of conservation increases. During the curing period spanning from 7 d to 14 d, the rise in fracture energy for the (SCECC) specimens surpasses the increase observed in the subsequent curing period, from 14 d to 28 d. The primary factor contributing to this observation is that, during the initial short curing period, the hydration reaction of the cement-based material is more intense. Consequently, the production of hydration products occurs at a faster rate during this timeframe. This rapid generation of hydration products leads to continuous pore filling within the cement-based material. As a result, the resistance of the material is significantly enhanced, particularly in terms of its ability to propagate cracks. This phenomenon is indicative of the crucial role played by the early stages of curing in promoting a more robust and crack-resistant cement-based material.

There is an escalation in tandem with the augmentation of PVA fiber volume fraction, demonstrating the influence of PVA fiber on fracture energy is substantial. This effect culminates in a maximum fracture energy of approximately 1973.70 J/m^2^ at the 28-day age. The fracture energy of the specimen with a PVA fiber volume fraction of 2.300% experiences a remarkable surge, increasing by 1120.95% in comparison to the specimen with a PVA fiber volume fraction of 0%. It can be attributed to the inherent capability of the fiber to impede the formation of microcracks and effectively hinder the propagation of cracks within the material [46].

### 3.3. Splitting Tensile Test Results Analysis

**Figure 13:**
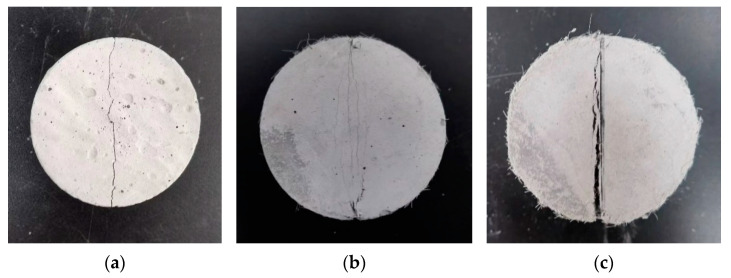
Failure mode of the splitting tensile test specimen: (**a**) *V_f_* = 0; (**b**) *V_f_* = 1.150%; (**c**) *V_f_* = 2.300%.

Figure 13 shows the failure mode of the splitting tensile test piece. Comparing the damage of split specimens with different fiber volume fractions, a specimen without fiber reinforcement splits with the first crack. Until a huge crack runs through the specimen, leading to the damage of the specimen. Before the damage to the specimen, many small cracks will appear around the main crack for the P-1.150 specimen. They are symmetrically distributed on both sides of the main crack, forming a more obvious multi-crack cracking phenomenon. As the fiber volume fraction continues to increase to 2.300%, more cracks appear in the splitting specimens during the loading process. The crack width is larger, the cracks appear in a wider range, and the multi-crack cracking phenomenon becomes more obvious.

**Figure 14:**
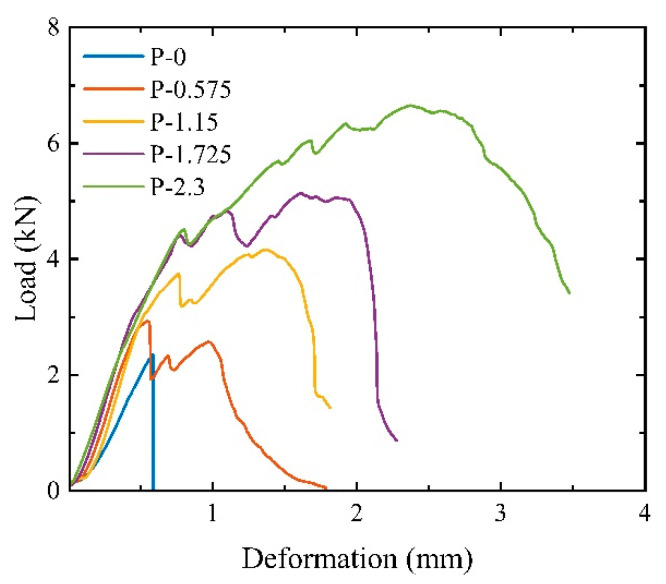
Splitting tensile load–deformation curves of SCECC.

Figure 14 shows the load–deformation curves of the SCECC splitting tensile specimen. As can be seen from the figure, the SCECC splitting load–deformation curve consists of five stages, which are the compacting, elasticity, crack formation, strain-hardening, and failure stages.

In the compacting stage, the press head continuously adjusts its contact position with the specimen until it is in full contact with the specimen. Due to the stress concentration on the contact surface between the press head and the specimen, the load–deformation curve appears significantly concave. In the elastic stage, the load–deformation curve shows good linear growth, and the SCECC matrix bears the main load. No cracks are formed on the surface of the specimen at this stage until the initial cracking point is reached. In the crack formation stage, where the first crack is formed, the deformation of the specimen grows rapidly and the load–deformation curve deviates from its original trajectory. In the strain-hardening stage, when the load reaches the yield load of the SCECC matrix, the deformation growth rate further accelerates, and the slope of the curve decreases rapidly. Due to the bridging effect of PVA fiber at the crack inhibiting crack expansion, the tensile stress at the crack continues to rise. And the load–deformation curve is “wavy”, showing obvious strain-hardening characteristics. In the failure stage, the cracks continue to expand, and the bridging capacity of the fibers gradually fails. The PVA fibers are pulled out or broken, the bearing capacity of the specimen decreases significantly, and splitting failure occurs.

As shown in Figure 14, the volume fraction of PVA fiber has a greater impact on the splitting load–deformation curve of the SCECC specimen. After the SCECC matrix specimen without PVA fiber enters the crack formation stage, the effective cross-section for transmitting tensile stress is reduced and there are no fibers to carry the tensile. This resulted in the specimen being rapidly destroyed. After adding PVA fiber, due to the bridging effect of the fibers across the cracks, the tensile capacity of the splitting surface after cracking can continue to increase. The greater the fiber content, the greater the increase in peak load, and the load–deformation curve shows different drop characteristics. When the fiber volume fraction reaches 0.575%, the curve drops significantly after the specimen reaches the peak load. However, due to the presence of PVA fiber, when the load drops to about 65% of the peak load height, a “pseudo-strain-hardening” phenomenon occurs. Due to the small fiber content, the tensile-bearing capacity of the split surface continues to decrease after a slight increase and still shows a certain degree of brittleness. When the fiber volume fraction reaches 1.115%, after passing through the crack formation stage, the load–deformation curve shows a slight drop and then an upward trend, reaching a peak after rising beyond the yield load. When the fiber volume fraction is 1.725%, the load–deformation curve is similar to the curve when the fiber volume fraction is 1.115%, and the load and deformation are larger. Compared with 1.725%, the linear relationship of the load–deformation curve of the specimen with fiber volume fraction reaching 2.300% is good before the yield load. After the yield load, the deformation growth rate accelerates and the slope of the load–deformation curve decreases rapidly, due to the PVA fiber at the crack. The principle of the bridging effect is like implanting many micro enhancers into the SCECC matrix. The fibers continue to bear the tensile force. The load–deformation curve rises significantly, showing strain-strengthening characteristics. The maximum load is the peak load. In summary, the addition of PVA is sufficient to change the failure mode of cement-based composite materials from brittleness to ductility, with excellent energy absorption and impact toughness [17].

The strength and the corresponding compressive deformations of splitting tensile specimens at 28-day age are listed in Table 5. It can be seen from Table 6 that before the yield and fiber pull-out stages since the load is mainly borne by the ECC matrix, the PVA fiber volume fraction has little effect on the tensile strength and compression deformation of the specimen at the initial crack point.

**Figure 15:**
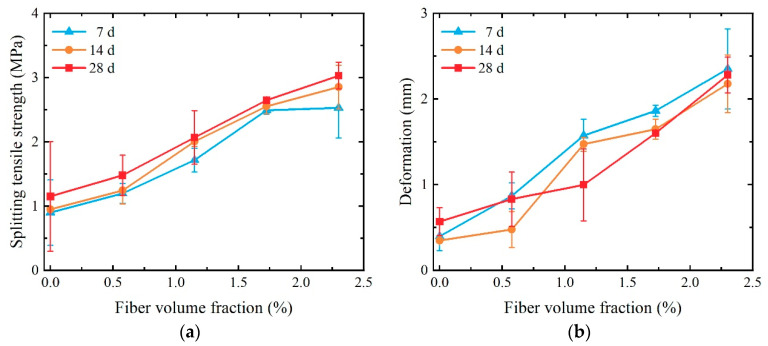
Splitting tensile strength and compression deformation of SCECC: (**a**) splitting tensile strength; (**b**) compression deformation.

Plot the relationship between split tensile strength and compression deformation on a line graph, as illustrated in Figure 15. Observing the figure, the increase in the volume fraction of PVA fibers, upon entering the strain-hardening stage, leads to an escalation in the bridging stress of the fibers at the crack. Consequently, there is a substantial enhancement in both the split tensile strength and compression-compression deformation of the SCECC specimens. At 28-day age, for instance, when the volume fraction of PVA fibers is below 0.575%, the fibers exhibit minimal bridging effects and lack the strength to withstand the tensile forces generated by matrix cracking to the extent that the specimen fractures at its weakest point during the crack formation stage. Hence, the first cracking strength and compression deformation of the specimen at the point of initial cracking are equivalent to the splitting tensile strength and compression deformation, respectively. When the volume fraction of PVA fibers is equal to or exceeds 1.150%, the fibers exhibit a robust bridging effect, resulting in split tensile strengths for the specimens that surpass their first cracking strength. When the fiber volume fraction increases from 1.150% to 2.300%, the splitting tensile strength and compressive deformation at the peak point of the specimen increase compared to the strength and deformation at the initial cracking point of 102% and 365%, respectively.

The tensile strength of SCECC can reach 3.03 MPa, which is close to the strength of ECC [43] but lower than that of UHDCC [42]. This indicates that SCECC does not perform well enough in the tensile field.

### 3.4. Evaluation of the Mechanical Properties in Different Groups of SCECC

**Figure 16:**
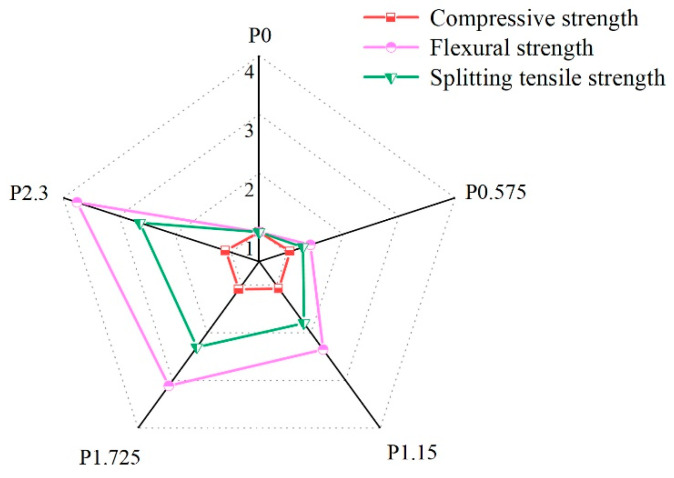
Mechanical properties of PVA fiber reinforced the vertical coordinates in the graphs representing the multiplicative increase in compressive, three-point flexural, and split tensile strengths of the various specimen groups relative to the P-0 group.

Figure 16 depicts the influence of (PVA) fiber volume fraction on the mechanical properties of (SCECC) at the 28-day age. The figure clearly illustrates the substantial impact of PVA fibers on all three strengths of SCECC. Notably, the strength of the material demonstrates a proportional increase with the corresponding rise in fiber volume fraction. At a fiber volume fraction of 2.300%, the compressive strength, three-point bending strength, and split tensile strength of the specimen exhibit the most significant improvements. Specifically, there are increases of 9.61%, 275.30%, and 34.26%, respectively, in comparison to the specimen without fibers. This observation underscores the positive correlation between PVA fiber content and the enhancement of mechanical properties in SCECC.

## 4. Conclusions and Future Prospective

The present study introduced an ECC material fabricated from local materials. A series of tests were made to explore the mechanical properties of the material and to give a preliminary indication of its main characteristics.

(1) SCECC exhibits a pronounced early strength. The compressive strength of the 7-day specimen can achieve more than 78.50% of that observed in the 28-day specimen. Furthermore, an increase in the fiber volume fraction corresponds to an elevated specimen load-carrying capacity and compression deformation; however, this growth rate decreases with an increasing fiber volume fraction.
(2) The damage mode of SCECC changed from brittle to ductile with the addition of a certain volume fraction of PVA. The stress–strain curve and fracture energy obtained from the three-point bending test demonstrate the high toughness of SCECC. As the fiber volume fraction increases, the flexural strength and fracture energy of the material tend to rise significantly, and the maximum flexural strength and fracture energy of the 28-day specimen could reach 13.43 MPa and 1973.70 J/m^2^, respectively.
(3) The properties of the fibers, which include delaying microcrack formation and preventing crack propagation, contribute significantly to the increased deformability of the material. The inhibition of cracks by fiber became more pronounced after the volume fraction of PVA fiber exceeded 1.150%. At the splitting tensile test, the PVA fibers spanning the cracks persist in carrying and transmitting tensile forces through a robust bridging action, imparting a notable strain-hardening characteristic to the specimen during the split tensile test.

In addition, this study has some limitations that need to be addressed and explored in future research:

(1) In the follow-up research, the influence of different coral sand aggregate gradation and freshwater mixing on SCECC will be further analyzed theoretically and experimentally to provide a theoretical basis for SCECC design.
(2) SCECC can meet general engineering needs, but its compressive and tensile strengths still need to be improved. In future research, consideration should be given to improving the defects of SCECC in these two aspects by changing the mixing ratio.

## Figures and Tables

**Table 1:** Mix proportions (ratio in weight).

	Cement	Fly Ash	Coral Sand	Seawater	SP	PVA Fiber
P-2.300	1	0.43	0.5	0.5	0.00123	*V_f_* = 2.300%
P-1.725	1	0.43	0.5	0.5	0.00123	*V_f_* = 1.725%
P-1.150	1	0.43	0.5	0.5	0.00123	*V_f_* = 1.150%
P-0.575	1	0.43	0.5	0.5	0.00123	*V_f_* = 0.575%
P-0	1	0.43	0.5	0.5	0.00123	*V_f_* = 0%

P = PVA fiber, SP = superplasticizer, *V_f_* = volume fraction of fiber.

**Table 2:** Chemical composition of coral sand.

Component	Na_2_O	MgO	Al_2_O_3_	SiO_2_	SO_3_	K_2_O	CaO	TiO_2_	Fe_2_O_3_	SrO
Mass fraction/wt%	0.46	0.5	2.43	6.06	0.43	0.35	85.25	0.29	2.07	1.91

**Table 3:** Aggregate grading parameters (%) and fineness modulus.

1.25–2.5 mm	0.63–1.25 mm	0.315–0.63 mm	0.16–0.315 mm	Fineness Modulus
5	15	65	15	2.1 (fine sand)

**Table 4:** Composition of artificial seawater.

Component	NaCl	CaCl_2_	MgSO_4_	MgCl_2_
Content (mmol·L^−1^)	445.20	17.22	51.68	64.82

**Table 5:** Physical and mechanical properties of PVA fiber.

Density (g·cm^−3^)	Tensile Strength (MPa)	Elastic Modulus (GPa)	Diameter (mm)	Length (mm)
1.2	1620	42.8	0.039	12

**Table 6:** Splitting tensile test date of SCECC.

	First Cracking Strength (MPa)	First Cracking Deformation (mm)	Splitting Tensile Strength (MPa)	Compressive Deformation (mm)
P-2.300	1.50	0.49	3.03	2.28
P-1.725	1.48	0.47	2.65	1.60
P-1.150	1.38	0.47	2.07	1.00
P-0.575	1.33	0.58	1.48	0.83
P-0	1.15	0.57	1.15	0.57

## Data Availability

Data are contained within the article.
